# Pressure-induced spin transition and site-selective metallization in CoCl_2_

**DOI:** 10.1038/s41598-019-41337-4

**Published:** 2019-04-01

**Authors:** Jose A. Barreda-Argüeso, Lucie Nataf, Fernando Aguado, Ignacio Hernández, Jesús González, Alberto Otero-de-la-Roza, Víctor Luaña, Yating Jia, Changqing Jin, Bongjae Kim, Kyoo Kim, Byung I. Min, Wilhem Heribert, Andrew P. Jephcoat, Fernando Rodríguez

**Affiliations:** 1 Universidad de Cantabria, MALTA Consolider Team - DCITIMAC, Santander, 39005 Spain; 2 Synchrotron SOLEIL, L’Orme des Merisiers, St Aubin BP48, 91192 Gif-sur-Yvette cedex, France; 3 Universidad de Oviedo, Departamento de Química Física y Analítica, Oviedo, 33006 Spain; 4 Institute of Physics, Beijing National Laboratory for Condensed Matter Physics, Chinese Academy of Sciences, Beijing, 100190 China; 5 Pohang University of Science and Technology, Department of Physics, PCTP, Pohang, 37673 Korea; 6 Kunsan National University, Department of Physics, Gunsan, 54150 Korea; 7 Pohang University of Science and Technology, Max Planck POSTECH/Hsinchu Center for Complex Phase Materials, Pohang, 37673 Korea; 8 Diamond Light Source Ltd, Chilton, Didcot, Oxfordshire OX11 0DE United Kingdom; 9 Okayama University, Institute for Planetary Materials, Yamada 827, Misasa, Tohaku, Tottori, 682-0193 Japan

## Abstract

The interplay between spin states and metallization in compressed CoCl_2_ is investigated by combining diffraction, resistivity and spectroscopy techniques under high-pressure conditions and *ab-initio* calculations. A pressure-induced metallization along with a Co^2+^ high-spin (S = 3/2) to low-spin (S = 1/2) crossover transition is observed at high pressure near 70 GPa. This metallization process, which is associated with the *p*-*d* charge-transfer band gap closure, maintains the localization of 3*d* electrons around Co^2+^, demonstrating that metallization and localized Co^2+^ -3*d* low-spin magnetism can coexist prior to the full 3*d*-electron delocalization (Mott-Hubbard *d-d* breakdown) at pressures greater than 180 GPa.

## Introduction

Pressure-induced structural phenomena have received considerable attention in transition-metal (*M*) dihalides and oxides *MX*_2_ (*X*: Cl, Br, F, O) due to their ample and subtle polymorphism^1–20^ and their intriguing electronic properties associated with changes of metal coordination, spin state, and/or insulator-metal transition. Most *MX*_2_ are antiferromagnetic Mott insulators^21^ that present strong *d-d* electron-electron correlation^22^. The breakdown of the Mott-Hubbard *d-d* or charge-transfer *d-p* electron correlation leading to a metallization, concurrent with a collapse of magnetism via electron delocalization, is the typical pressure-induced electronic/magnetic behavior. These phenomena involve external pressures that induce a large crystal-field strength at *M*, causing the high-spin to low-spin (HS-LS) transition^1,23–25^. In fact, spin crossover (SCO) is often portrayed as a trigger for metallization either by volume collapse^22,25^, or as a result of the ground-state change from HS to LS^22–24^. Nevertheless, the coupling between structure, spin state, and electron delocalization (Mott-Hubbard metal-insulator transition) governing the electronic properties in *MX*_2_ requires clarification. The access to both electronic ground and excited states via optical spectroscopy at high pressure, combined with the modelling of the electronic properties through *ab initio* calculations and precise crystal structure determination and resistivity measurements, can provide a definitive description of electronic phenomena in compressed *MX*_2_ systems.

Pressure-induced transformations in *MX*_2_ involve a large variety of energetically-equivalent dense structures, which are characterized either by an increase of the *M* coordination number^11,20^, or by stacking up dense *MX*_2_ layers keeping the metal coordination^14,26^, depending on the metal/ligand ionic radii ratio. Due to their simple structure, phase diagrams of crystals in the *MX*_2_ family show common features regarding the coordination polyhedra and the stacking sequence. This distinct structural behavior has important implications in their electronic properties, which are substantially modified upon compression. Besides, these studies of *MX*_2_ under pressure are of importance in geophysics to understand polymorphism in the Earth’s interior, where SiO_2_ plays an important role^27–29^.

CoCl_2_ is an attractive system for studying combined structural and electronic effects because the octahedral coordination of Co^2+^ (3*d*^7^) is thought to be stable in a wide pressure range^13–15,26,30^, which enables us to exclude the physics coming from the coordination number changes. Besides, this system has a relatively small energy difference between its HS and LS phases^31^, and the SCO of the system can be observed in a very  accessible pressure regime. Furthermore, it is the member of the *M*Cl_2_ series (*M*: Cr, Mn, Fe, Co) where SCO is expected to take place at the lowest pressure. In addition, SCO phenomena involving transition-metal ions with 3*d*^7^ electronic configuration are very scarce. Thus, this system can be a good model system to explore the various origins of SCO and to give answer to questions such as whether the pressure-induced LS states (*t*^6^*e*^1^ configuration) originate from large crystal-field effects enhanced by the Jahn-Teller effect associated with LS configuration, or from the metallization, which can be induced either by the *p-d* charge-transfer gap closure or breakdown of *d-d* Mott-Hubbard correlation.

Here we report on the stability of the Co^2+^ coordination under compression in CoCl_2_, and the relationship between SCO, crystal structure, and metallization. In order to achieve these goals we perform a combined experimental and theoretical study using optical absorption, Raman spectroscopy, X-ray diffraction (XRD) and resistivity under pressure, and first-principles Density Functional Theory (DFT) calculations.

## Results

**Figure 1:**
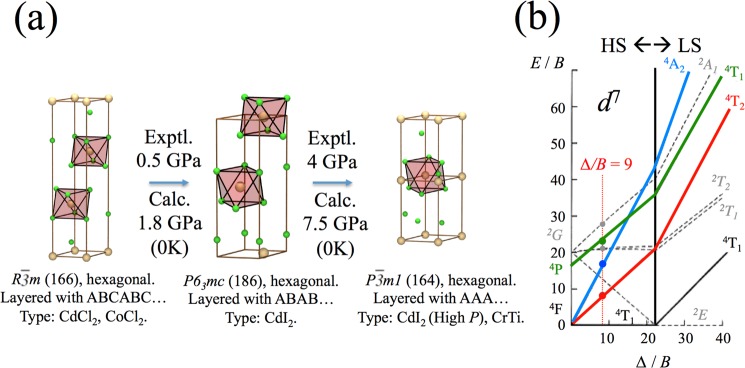
(**a**) Structural phase-transition sequence in CoCl_2_ under high-pressure conditions determined by density-functional-theory calculations and confirmed experimentally by x-ray diffraction. (**b**) Simplified Tanabe-Sugano diagram for octahedral Co^2+^ (3*d*^7^) showing the effect of pressure (i.e. Δ/*B*) on the excited state energies, and HS-LS crossover (Δ/*B* = 21). See also Supplementary Figs S1–S9.

The Co^2+^ (3*d*^7^) Tanabe-Sugano diagram describing the electronic states’ energy in terms of the octahedral crystal field (in Racah parameter *B* units, Fig. 1) shows that, for CoCl_2_ (*B* ≈ 80 meV at 50 GPa^15,30^), the HS-LS (^4^T_1_, S = 3/2 → ^2^E, S = 1/2) transition should occur at Δ_*SCO*_ = 1.7 eV. Importantly, the LS state may be affected by a strong Jahn-Teller effect, providing an additional lattice relaxation energy which, in principle, could reduce the SCO crystal-field strength triggering metallization at unexpectedly low pressure^19,30^. Alternatively, high pressure could, in turn, suppress the Jahn-Teller distortion causing the HS-LS to occur at higher pressures than expected, or even disappear if CoCl_2_ transforms into a fluorite-type structure (*d*^3^-like Co^2+^)^20^.

XRD shows that CoCl_2_ exhibits nearly-degenerate layered structures at ambient conditions. The CoCl_2_ pressure-induced phase transition sequence as determined experimentally with support of DFT calculations is indicated in Fig. 1. The three represented layered structures are more stable than the rutile, cotunnite, and fluorite phases at all pressures up to 100 GPa. This result, which is confirmed by both single-crystal and powder XRD experiments at high pressure, demonstrates the stability of the hexagonal layered structure of CoCl_2_ and thus the Co^2+^ sixfold coordination in a wide pressure range (0–60 GPa), in a way similar to FeCl_2_^14^ and MgCl_2_^26^ and contrary to CoF_2_^10,20^. The stability of the given CoCl_2_ structures, which involve the different packing sequence of layers of face-sharing CoCl_6_ octahedra, is a consequence of the subtle competition of inter-layer van der Waals interactions. The CoCl_2_ equation-of-state can be phenomenologically described by two Murnaghan’s equations: one above and one below 14 GPa (see Supplementary Figs S1–S3).

**Figure 2:**
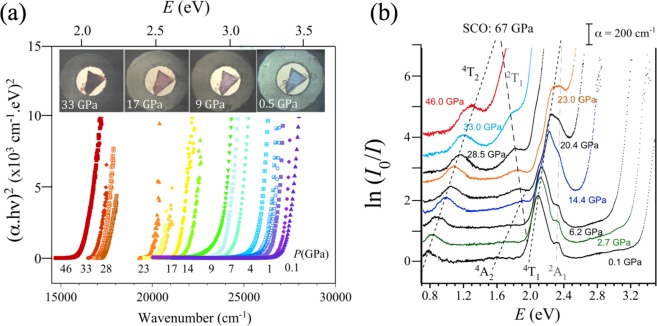
Pressure dependence of the optical absorption spectrum of CoCl_2_. (**a**) Variation of the charge-transfer absorption threshold in the 0–50 GPa transparency region. The pictures show the piezochromism of CoCl_2_. (**b**) Pressure dependence of the Co^2+^ crystal-field absorption peaks. Peak labeling follows the Tanabe-Sugano diagram of Fig. 1.

Figure 2 shows the pressure dependence of the optical absorption spectra of CoCl_2_ around the charge-transfer band gap (a) and in the sub-gap Co^2+^ *d-d* crystal-field region (b). Besides the gap energy, these spectra allow us to determine the excited-state electronic structure in the transparency window of CoCl_2_ (≈50 GPa). At ambient conditions, the main sub-gap absorption peaks within the *D*_3*d*_ (nearly *O*_*h*_) CoCl_6_^4−^ octahedron^7,8^ correspond to crystal-field transitions ^4^T_1_(F) → ^4^T_2_(F), ^4^A_2_(F), and ^4^T_1_(P) and are located at 0.79, 1.66 and 2.10 eV, respectively. In terms of the Tanabe-Sugano diagram for *d*^7^ ions (Fig. 1b)^32,33^, the transition energies at ambient pressure correspond to Δ = 0.87 eV and *B* = 97 meV with Δ/*B* = 9.0 (see Supplementary Table S1)^15,30,32^. According to this diagram the crystal-field strength required to induce the SCO is (Δ/*B*)_*SCO*_ = 21. Interestingly, the SCO also involves crossing of the ^4^T_2_(F) and ^2^T_1_(G) excited states, hence these states, which are well observed by optical absorption, can be used to efficiently probe the HS-LS transition.

**Figure 3:**
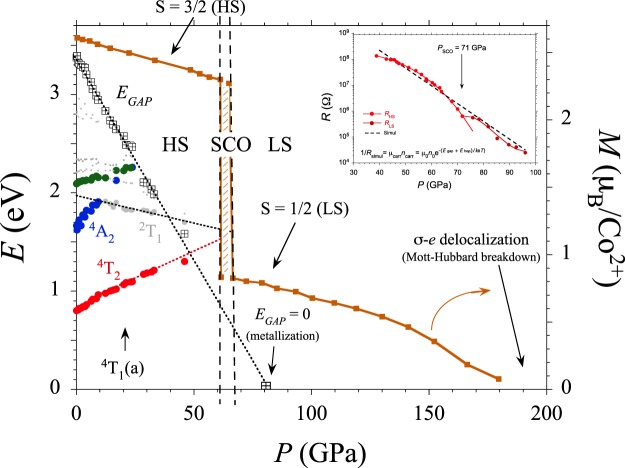
Variations of the Co^2+^ crystal-field state energy of the ^4^T_1_(a) → Γ_*i*_ transition and of the charge-transfer band gap energy of CoCl_2_ (*E*_*GAP*_) with pressure. The color of the experimental points (Γ_*i*_) is the same one employed for representing the energy of Γ_*i*_ state in the Tanabe-Sugano diagram of Fig. 1. Note that the HS-LS crossover corresponds to the ^4^T_2_(F) ↔ ^2^T_1_(G) crossing point, and the *p-d* band gap closure to *E*_*GAP*_ = 0 (metallization). The continuous curves in brown color are the calculated Co^2+^ local moment within DFT + U method keeping the *P*$\overline{3}$ *m1* phase in the whole pressure range. The HS-LS transition and *p-d* band gap closure take place at 67 and 80 GPa, respectively, whereas the Mott-transition (*σ*-electron delocalization) in LS is found for *P* > 180 GPa (detailed information in Supplementary Figs S4–S7). The inset shows the variation of the electrical resistance of CoCl_2_ with pressure, *R*(*P*), in a semilog plot. It must be noted that *R*(*P*) behaves differently below and after 71 GPa indicating SCO transition. The change of slope above 80 GPa unveils the metallization onset. Linear dashed line corresponds to the least-square fit to *R*(*P*) taking the linear variation of *p-d* charge-transfer band gap, *E*_*GAP*_, obtained by optical absorption, and a thermal activated hoping term, *E*_*hop*_(*P*) associated to the carrier mobility (see also Supplementary Fig. S12).

The variation of the absorption spectra with pressure shows that the band gap energy decreases linearly with pressure at a rate of −43 meV/GPa (Fig. 3). Such a large shift is responsible for the intense piezochromism exhibited by CoCl_2_ (Fig. 2a). The pressure-induced redshift of the bandgap follows a quadratic dependence with the crystal volume yielding gap closure at *V* = 17.5 Å^3^/Co (*V*/*V*_0_ = 0.56) – i.e. 80 GPa– (see Supplementary Fig. S5(c)). This redshift is produced by the hybridization enhancement of the Cl^−^-*p* and Co^2+^-*d* orbitals with pressure which in turn causes a broadening of the mainly 3*p*- and 3*d*-orbital valence band and an energy decrease of the mainly 3*d*-orbital intermediate band both reducing the *p-d* charge-transfer bandgap. DFT reproduces the decrease in the band gap of HS state (*P* < 67 GPa) reasonably well (see Supplementary Fig. 6(c)). The plots of the electron band and density of states certainly show a clear energy delocalization of the *d*-orbital manifolds with pressure yielding band broadening (see Supplementary Fig. S7(c,d)). Concurrently, the increasing crystal-field energy as obtained from the optical spectra, and the reduction of *B* from 97 to 82 meV in the 0–50 GPa range yield a Δ/*B* variation from 9.0 to 18.5, which implies an almost doubled splitting between *e* and *t*_2_ orbitals, Δ, from 0.87 to 1.52 eV (see Supplementary Table S1 and Fig. S5).

**Figure 4:**
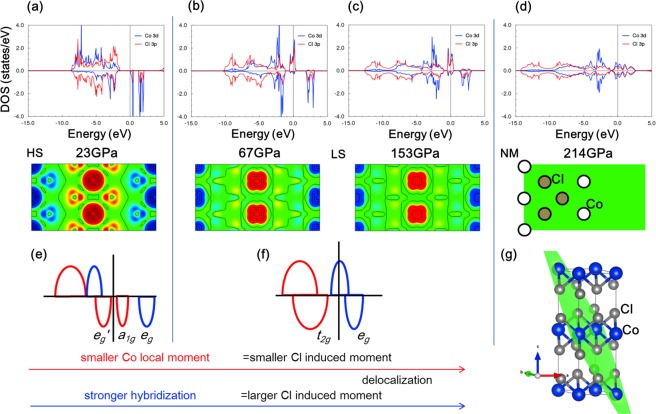
Total energy DFT + U calculations performed on CoCl_2_ (space group *P*$\overline{3}$ *m1*) using a Coulomb correlation energy, *U* = 3 eV. Top panels show the electron DOS projected on the Co^2+^ 3*d* (blue) and Cl^−^ 3*p* (red) orbitals at 23 (HS), 67 (LS), 153 (met) and 214 (deloc) GPa (**a**–**d**). The corresponding spin density around the Fermi level on the (111) plane is shown in panels below with red and blue denoting up and down spin density. A schematic Co^2+^ 3*d* bands associated with *t*_2*g*_ (*e*_*e*′_, *a*_1*g*_) + *e*_*g*_ in blue and red colors, respectively, illustrates the HS and LS states (**e**,**f**). It must be noted that spin density in LS is significantly localized around Co^2+^ whereas it is more delocalized around Cl^−^ in spite the electronic Fermi levels have about the same contributions from Cl^−^-*p* and Co^2+^-*d* orbitals (detailed information in Supplementary Figs S7–S10). Note that above 150 GPa, calculated pressures by DFT + U are overestimated as the Coulomb correlation energy was fixed to *U* = 3 eV. Indeed pressure should be corrected by about 20% in that range if *U* decreases by 20% due to ultra-high pressure effects. The lattice structure of CoCl_2_ is depicted in (**g**). Note that the green plane is (111) plane for spin density plot.

As Figs 2 and 3 show, the variation of the absorption spectrum and its associated peak energies with pressure reveal that Co^2+^ has a HS state in the crystal transparency range. However, extrapolating the linear dependence of the transition energies with pressure we obtain a HS to LS [^4^T_1_(F) ↔ ^2^E(G)] transition at 67 GPa. It is worth noting that the SCO is observed in the DFT + U results using a Coulomb correlation energy *U* = 3 eV (Figs 3 and 4). As indicated in the Methods section, this method cannot capture the evolution of the Coulomb correlation parameter upon volume changes and could underestimate the SCO pressure for high pressure regime^31,34,35^. However, the essence of the electronic and magnetic properties before and after the transition is valid. The spectroscopic determination of the 3*d*-electron structure together with the DFT estimates make CoCl_2_ a reference system to validate theories dealing with SCO phenomena and metallization processes in transition-metal systems^25,36–43^.

Figure 3 plots the calculated magnetic moment as a function of pressure. According to DFT calculations, the Co^2+^ magnetic moment abruptly decreases from HS, *μ*_*eff*_ = 2.6 *μ*_*B*_, to LS, *μ*_*eff*_ = 0.9 *μ*_*B*_, at around 67 GPa with a hysteresis of 8 GPa. The experimental SCO pressure and the crystal and electronic structures demonstrate that the Jahn-Teller coupling is not involved in the stabilization of the LS ground state, ^2^E. This contrasts with one of the hypotheses given elsewhere^30,31^, that the high-pressure conditions required for SCO could be relaxed by the strong Jahn-Teller effect in the LS ^2^E state down to 35 GPa if we consider a Jahn-Teller coupling similar to those measured in the CuCl_6_ system^44^. The lack of a HS-LS transition at ≈35 GPa in CoCl_2_ indicates that the Jahn-Teller effect is unable to distort the Co^2+^ environment in the severe high-pressure conditions required for SCO.

The pressure dependence of the optical gap, *E*_*GAP*_, allows us to infer that the *p-d* charge-transfer gap closure (metallization) takes place at 80 GPa. This result is confirmed by electrical resistance measurements under pressure (inset of Fig. 3 and Supplementary Fig. S12). Its pressure dependence *R*(*P*) unveils two distinct regions corresponding to HS and LS states. The associated SCO pressure, *P*_*SCO*_ = 70 GPa, is close to that derived from optical absorption. Interestingly, the progressive decrease of *R*(*P*) in LS shows a change of slope for *P* > 80 GPa indicating the metallization onset. Spin density- and DOS simulations indicate that the charges are mainly localized at the Co^2+^ site and small *p-d* hybridized ones can be observed at Cl^−^ sites for HS. For *P* > 67 GPa (LS) a progressive decrease of the localized charges at Co^2+^ occurs due to hybridization increase. However, the hybridized spin density spreads out over the entire crystal for *P* > 80 GPa (*E*_*GAP*_ = 0) in the Cl^−^ plane, while it is strongly localized at Co^2+^, indicating that metallization mainly involves Cl^−^ sublattices rather than the Co^2+^ ones. Furthermore, a full electron delocalization is completed for *P* > 180 GPa (Fig. 4). This result is noteworthy since it correlates two distinct electronic features: (1) the insulator-to-metal transition involves *p-d* charge-transfer states and can be induced under compression in close proximity right after the HS-LS transition; (2) Mott-Hubbard *d-d* electron breakdown should occur for *P* > 180 GPa. Thus, 3*d*(*e*)-electrons still keep their local character at the band-gap closure, albeit pressure-induced progressive delocalization occurs within LS ground state up to approximately 180 GPa, at which delocalization process is completed (Figs 3 and 4).

## Conclusions

In summary, with various types of experimental and theoretical approaches, we have thoroughly analysed the physics of the pressure-induced spin-state transition and metallization phenomena in CoCl_2_. We have shown that the layered structure of CoCl_2_, and hence the Co^2+^ sixfold coordination, is stable in the 0–200 GPa range, in contrast to CoF_2_, whose high-pressure phases involve increasing coordination numbers (6 → 8 → 9). We demonstrate that pressure-induced metallization is associated with *p-d* charge-transfer band gap, closing at about 80 GPa. Although the HS-to-LS transition (67 GPa) can trigger insulator-to-metal transition, DFT calculations also show that after the SCO metallization Co^2+^ preserves the local character of the 3*d*-electrons and that Mott-Hubbard-electron breakdown takes place for *P* > 180 GPa in LS. In consequence, this work demonstrates that metallization with involvement of Cl^−^ planes and localized Co^2+^-3*d* LS magnetism can coexist prior to Mott-Hubbard breakdown in CoCl_2_. These results unveil the complex metallization mechanism of CoCl_2_ under compression with Cl^−^ and Co^2+^ layers exhibiting site-dependent electrical and magnetic behaviours. Especially, the intermediate phase with metallic magnetism is rarely observed in a system with local moment such as transition metal complexes. We believe these findings provide new insight into unforeseen electronic properties of multilayer 2D systems and highlight the importance of high-pressure studies as a route to novel electronic and magnetic phases.

## Methods

### Crystal structure: X-ray diffraction

Both single-crystal plates (100 × 80 × 30 *μ*m^3^) and powder of CoCl_2_ (Merck) were used for high-pressure experiments. CoCl_2_ crystallizes in the trigonal space group *R3m* at ambient conditions^45^. The evolution of the crystal structure with pressure was studied by x-ray diffraction (XRD) using the I15 beam station at the DIAMOND synchrotron under proposals 832, 1655 and 6078. The pressure was applied by means of Almax-Boehler and MALTA-type Diamond Anvil Cell (DAC). DACs were loaded with several Ruby spheres (10 *μ*m diameter) as pressure gauge^46^ using helium, silicone oil and paraffin as pressure transmitting media for powder and single crystal XRD experiments (see Supplementary Figs S1–S3).

### Optical absorption and Raman spectroscopy

**Figure 5:**
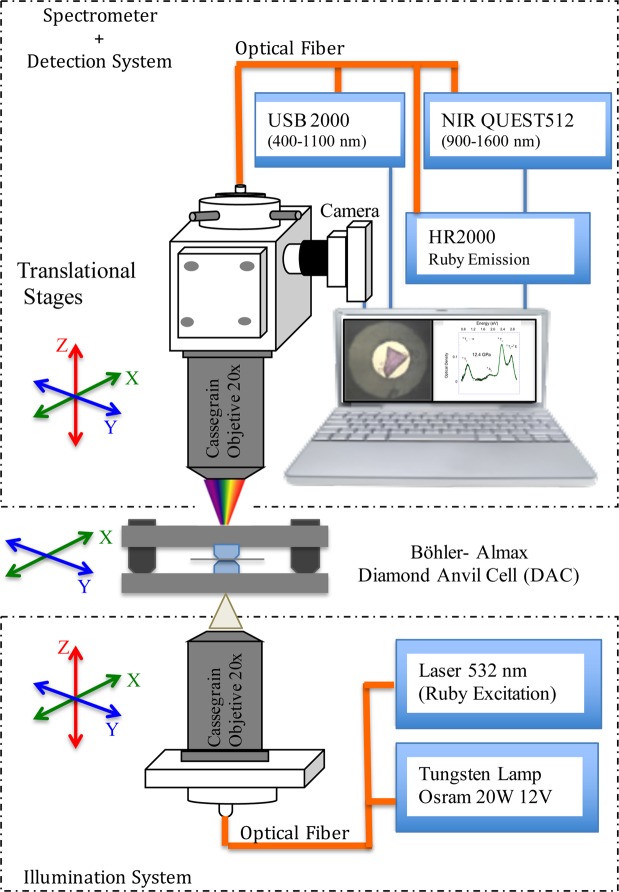
Schematic setup of the microscope adapted for optical absorption with attached diamond anvil cell [Barreda-Argüeso, J. A. and Rodríguez, F. (Patent PCT/ES2014/000049)].

Optical absorption and Raman experiments were performed on single-crystal plates (100 × 80 × 35 *μ*m^3^) of CoCl_2_. The optical spectroscopy experiments were carried out in membrane and Almax-Boehler DACs. 200-*μ* m-thick Inconel 625 gaskets were preindented to 40 *μ*m. 170-*μ* m-diameter holes were perforated with a BETSA motorized electrical discharge machine. The DAC was loaded with a CoCl_2_ single crystal and ruby microspheres (10 *μ*m diameter) as pressure probes^46^ using silicone oil as pressure-transmitting medium in an argon atmosphere inside a globe box to avoid sample hydration. Optical absorption under high-pressure conditions was performed on a prototype fiber-optics microscope equipped with two 20× reflecting objectives mounted on two independent x -y -z translational stages for the microfocus beam, and the collector objective and a third independent x -y -z translational stage for the DAC holder (Fig. 5). Optical absorption data and images were obtained simultaneously with the same device. Spectra in the UV-VIS and NIR were recorded with Ocean Optics USB 2000 and NIRQUEST 512 monochromators using Si- and InGaAs-CCD detectors, respectively.

Unpolarized micro-Raman scattering measurements were performed with a triple monochromator Horiba-Jobin-Yvon T64000 spectrometer in subtractive mode backscattering configuration, equipped with a Horiba Symphony liquid-nitrogen-cooled CCD detector. The 514.5-nm and 647-nm lines of a Coherent Innova 70 Ar^+^-Kr^+^ laser were focused on the sample with a 20× objective for micro-Raman, and the laser power was kept below 4 mW in order to avoid heating effects. The laser spot was 20 *μ*m in diameter and the spectral resolution was better than 1 cm^−1^. The Raman technique was used to check the sample structure through the characteristic first-order modes (A_1*g*_ and E_*g*_ in the trigonal *R*$\overline{3}$*m* CdCl_2_-type phase)^38^ as well as to determine structural phase-transition pressures (see Supplementary Figs S10 and S11 and Table S3). The Raman high-pressure experiments were performed on the same CoCl_2_ single crystals employed in the optical absorption measurements.

### Electrical measurements at high pressure

**Figure 6:**
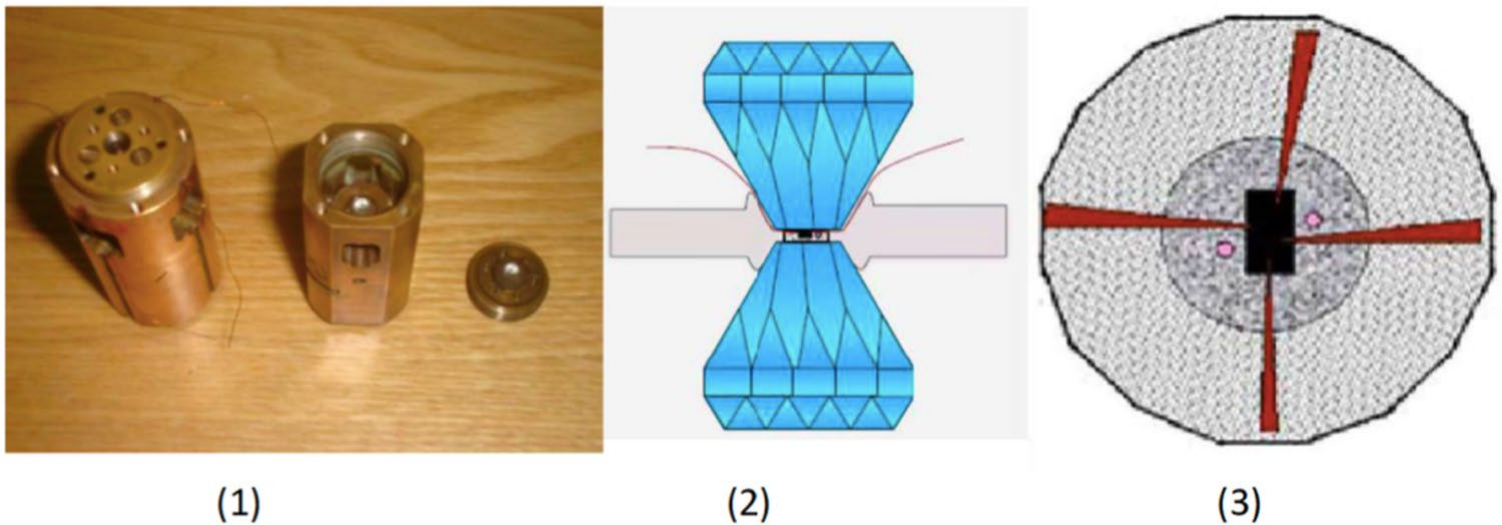
Setup for electrical resistance measuremets under high pressure conditions. (**1**) Piston-cylinder diamond anvil cell, (**2**) schematic view of opposite anvils pressing the CoCl_2_ sample, and (**3**) view of the four-points electrical contacts in the CoCl_2_ single crystal.

The electrical resistance measurement under pressure up to 96 GPa was performed using diamond anvil cell with solid transmitting medium NaCl (diamond’s culet diameter of 100 *μ*m). Gasket consists of T301 and the insulate layer is cBN. Pressure was determined by ruby fluorescence method at low pressure and the shift of diamond’s Raman peaks. Figure 6 shows a schematic view of the DAC.

### First-principles theoretical calculations

#### Density functional theory: crystal structure and phase transition

For the description of an accurate phase transition, we have performed total-energy calculations within the framework of dispersion-corrected Density-Functional Theory in the Projector Augmented Wave (PAW) and plane-waves (PW) formulation. The Perdew-Burke-Ernzerhof (PBE) exchange-correlation functional was employed coupled with the exchange-hole dipole moment (XDM) model dispersion correction (damping function parameters a_1_ = 0.0000 and a_2_ = 3.8036 Å) as implemented in Quantum ESPRESSO. The calculation parameters are: 6 × 6 × 6 Monkhorst-Pack k-point grid, 60 Ry plane-wave cutoff energy, 600 Ry density-cutoff energy and cold smearing with a smearing parameter of 0.05 Ry. Based on previous studies, we considered the following phases of CoCl_2_: cotunnite (*Pnma*, orthorhombic, Z = 4), CaCl_2_ (deformed rutile structure, *Pnnm*, orthorhombic, Z = 2), fluorite (*Fm*$\overline{3}$ *m*, cubic, Z = 1), CoCl_2_ (*R*$\overline{3}$ *m*, rhombohedral, ABCABC stacking), CdI_2_ (*P6*_3_ *mc*, hexagonal, ABAB stacking), *ω*-phase (*P*$\overline{3}$ *m1*, hexagonal, AAA stacking). All CoCl_2_ phases were calculated in a range of volumes encompassing the 0–100 GPa range and the internal degrees of freedom (atomic positions and cell shape) were relaxed at each volume.

#### Density functional theory: spin crossover and metallization

After clarifying structural phase transitions, we identified that the electronic and magnetic transitions occur in *P*$\overline{3}$ *m1* phase. Thus, employing the same symmetry, we investigated the SCO behaviors in detail. We further performed electronic-structure calculations within DFT + U scheme as implemented in Vienna Ab Initio Simulation Package (VASP)^47^. As for layered system, where the van der Waals interactions are important, frequently used generalized gradient approximation (GGA) functionals sometimes fails to predict the correct structural behaviors. We found van der Waals-corrected functions gives better description of the ground state volume such that the errors were 1.6% for many-body dispersion and 2.2% for Tkatchenko-Scheffler methods while D3 approach severely underestimates the volume by 8.2%^31,34,35^). From GGA^48^, we found that PBEsol overperforms PBE (3.8% vs. 8.1%) with accuracy similar to van der Waals approach, which enables us to choose PBEsol scheme with safety. Note that in our previous reports, PBEsol successfully explained the spin-state transition behaviors for CoCl_2_^31^. We also carefully tested various U parameters and found that *U*_*eff*_ = 3.0 eV fits best in describing the experimental transition behaviors. To obtain the pressure evolution of the electronic structure and magnetic properties, we fully relaxed the atomic positions until the atomic forces are less than 0.001 eV/Å for each volume point. Once the transition volume is found, we have cross-checked the results employing full potential full relativistic code FPLO^49^, and further analyzed its partial density of states (see Supplementary Figs S6–S9).

## Supplementary information

Supplementary Material

## Data Availability

All data generated or analysed during this study are included in this published article (and its Supplementary Information files).
