# Supplementary material for: Pressure-induced spin transition and site-selective metallization in CoCl2
Source: Sci Rep. 2019 Apr 1;9:5448. doi: 10.1038/s41598-019-41337-4 (PMC6443712; doi:10.1038/s41598-019-41337-4)
Supplement: Supplementary file 1 — Supplementary Material [file 41598_2019_41337_MOESM1_ESM.pdf]

Supplementary Material

Pressure-induced spin transition and site-selective metallization in $CoCl_2$.

Jose A. Barreda-Argüeso[1], Lucie Nataf[2], Fernando Aguado[1], Ignacio Hernández[1], Jesús González[1], Alberto Otero-de-la-Roza[3], Víctor Luaña[3], Yating Jia[4], Changqing Jin[4], Bongjae Kim[5,6], Kyoo Kim[7], Byung I. Min[5], Wilhem Heribert[8], Andrew P. Jephcoat[9], and Fernando Rodríguez[1,*]

Table of contents:

Topic	*Figure/Table (Matter)*	*Page*
X-ray diffraction	Figure S1 (powder)	S2
	Figure S2 (single crystal)	S3
	Figure S3 (Equation of State)	S4
Optical absorption	Figure S4 (Optical energies)	S5
	Figure S5 (Energy gap)	S6
	Table S1 (Spectroscopic parameters)	S7
First principles DFT+U	Table S2 (vdW calculations)	S8
	Figure S6 (Spin crossover)	S9
	Figure S7 (Density of States)	S10
	Figure S8 (DOS projection)	S11
	Figure S9 (Electron delocalization)	S12
Raman spectroscopy	Figure S10 (Spectra $\nu(P)$)	S13
	Figure S11 (Plot $\nu(V)$)	S14
	Table S3 (Grüneisen parameters)	S15
Electrical measurements	Figure S12 ($R(P)$)	S16

[*] E-mail address: rodriguf@unican.es (F. Rodriguez)

X-ray diffraction

Powder

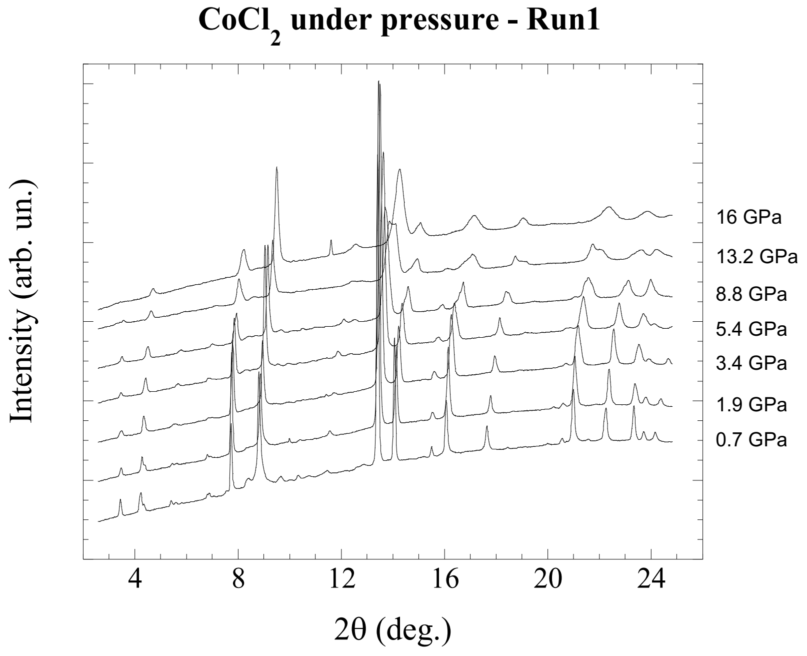

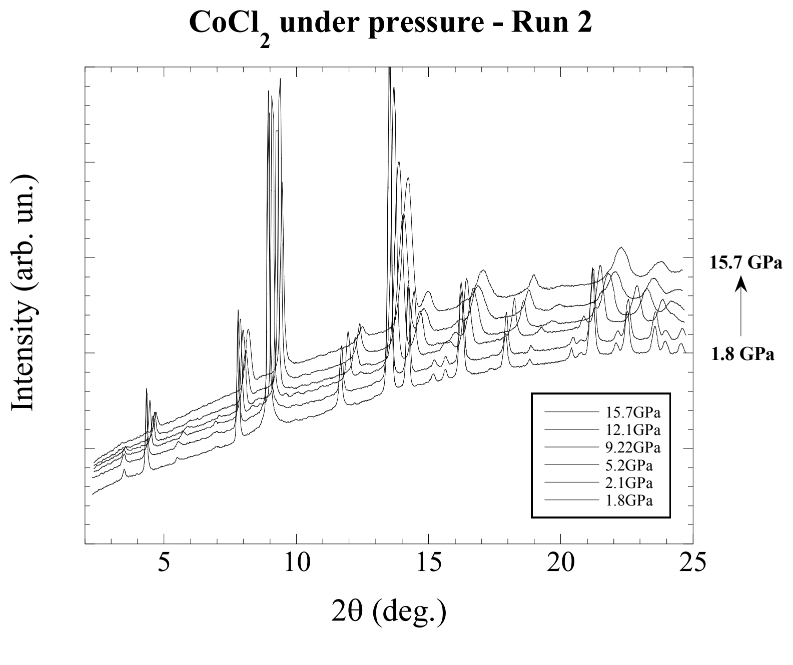

Figure S1. Pressure evolution of the $CoCl_2$ diffraction patterns in the 0-20 GPa range for two different runs using (a) silicon oil Run 1; and (b) helium Run 2 as pressure transmitting media. This procedure allows us comparison with the optical absorption and Raman spectroscopy studies carried out in $CoCl_2$ in this work.

Single crystal

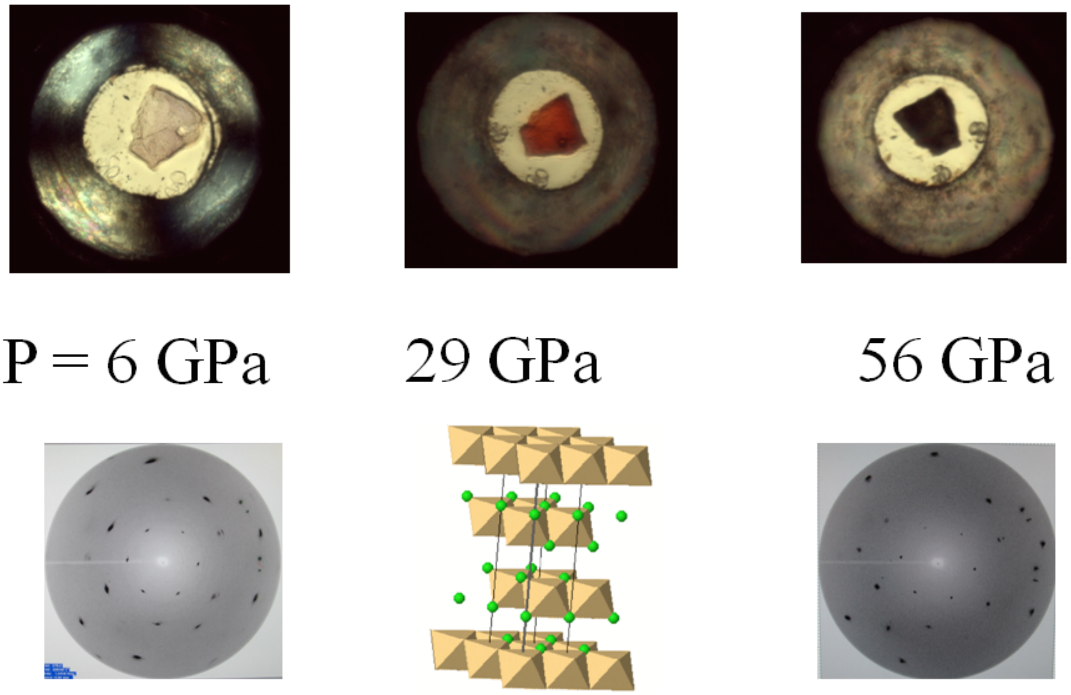

Figure S2. Pressure evolution of the (001)-oriented $CoCl_2$ single crystal diffraction patterns in the 0-60 GPa range. Note the color change upon pressure illustrating the transformation from insulating to semiconductor. The XRD pattern reflects the hexagonal crystal symmetry. Diffraction patterns and corresponding photographs were taken in downstroke.

Equation of State

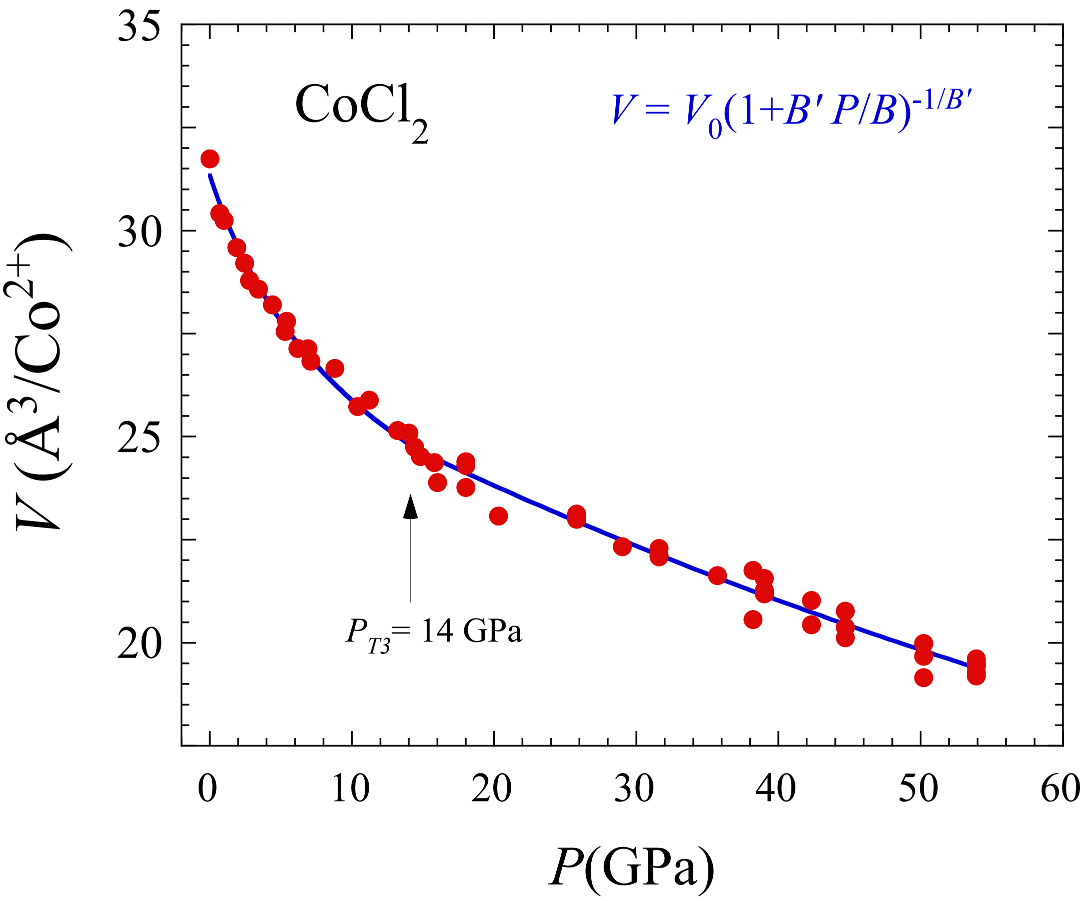

Figure S3. Pressure dependence of the hexagonal cell volume (Z = 2) of $CoCl_2$. The plot includes data from two powder XRD runs in the 0-20 GPa and one single-crystal run in the 0 – 60 GPa range. The blue line corresponds to the fit of two Murnaghan Equation of State to the experimental data $V(P)$ (one for $P < 14$ GPa [fit parameters: $V_0 = 31.34(15)$ Å^3; $B = 29.6(1.8)$ GPa; $B' = 5.5(7)$] and $P > 14$ GPa [fit parameters: $V_0 = 27.3(5)$ Å^3; $B = 140(30)$ GPa; $B' = 1(1)$]). The diffraction patterns above and below 14 GPa are consistent with a $P\bar{3}m1$ space group suggesting the occurrence of a second-order, isostructural phase transition.

Electronic structure derived from Optical absorption

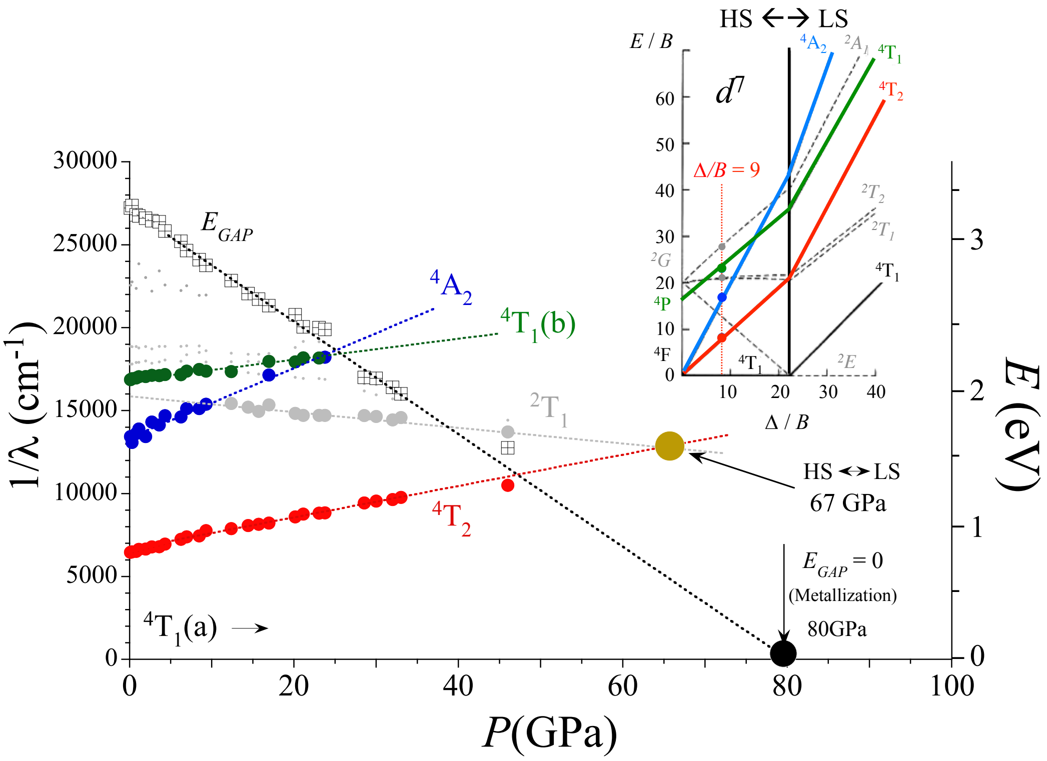

Figure S4. Pressure variations of the Co^{2+} crystal-field state energy in $CoCl_2$. The experimental points correspond to electronic transitions from the high-spin $^4T_1$(a) electronic ground state of Co^{2+} (O_h-symmetry notation) to the Γ_i excited state, and to the band gap energy, E_{GAP} as a function of pressure. The High-Spin-to-Low-Spin (HS-LS) and Charge-Transfer metallization (*p*-*d* band gap closure: $E_{GAP} = 0$) are found at a similar pressure: 67 and 80(10) GPa, respectively. The inset shows the Tanabe-Sugano energy level diagram for d^7 ions. The energy of the Γ_i state is represented as a function of the crystal-field splitting and are both given in units of the Racah parameter *B*. Color representing Γ_i state has been maintained in both figures. Experimental points at $\Delta/B = 9$ correspond to ambient pressure conditions. Note that the HS-LS spin crossover transition corresponds to the $^4T_2(F) \leftrightarrow {}^2T_1(G)$ crossing point in the Tanabe-Sugano diagram ($\Delta/B = 21$), and are marked in the two plots by an orange spot.

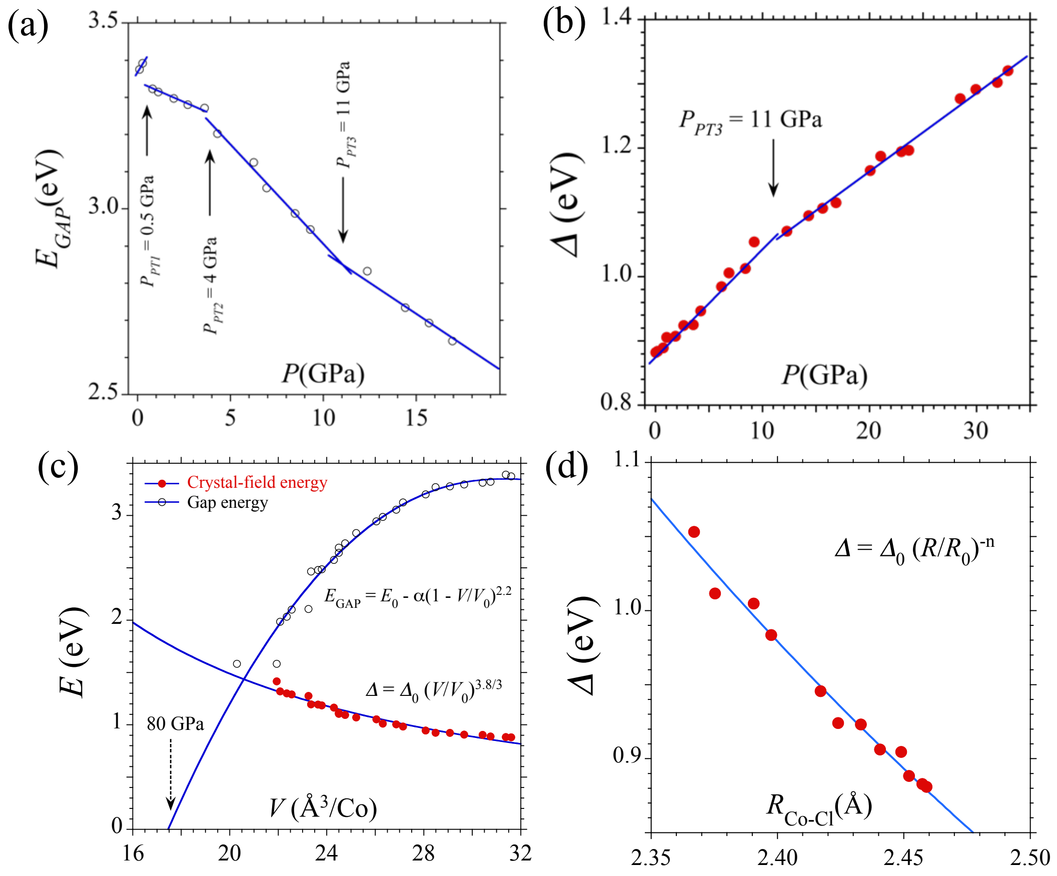

Figure S5. (a) Energy gap pressure dependence $E_{GAP}(P)$ in the low-pressure range. Discontinuities at 0.5 and 4 GPa are associated with structural phase transitions: *R3m* → *P6₃mc* → *P3m1*. (b) Pressure dependence of the crystal-field energy $\Delta(P)$ showing a change of slope at 11 GPa. This is probably related to the occurrence of a second-order, isostructural phase transition (*P3m1*), detected by XRD at 14 GPa (Fig. S3). (c) Volume dependences of $E_{GAP}(V)$ and $\Delta(V)$. Continuous lines are least-square fits to the given equations. Fit parameters are: $E_{GAP}(V_0) = 3.35(5)$ eV; α = 20(3) eV; $\Delta_0 = 0.84(5)$ eV; $V_0 = 31.34$ Å^3 per Co^{2+}, with exponents 2.2(2) and 3.8(2). (d) Co-Cl bond distance dependence of $\Delta(R_{Co-Cl})$ in the 0-20 GPa range. The line is the least-square fit to a potential function with parameters: $R_0 = 2.46(2)$ Å, $\Delta_0 = 0.88(5)$ eV; $n = 4.5(2)$. Note that $\Delta/\Delta_0 = (V/V_0)^{-1.3}$ in the 0-50 GPa range, significantly deviates from R^{-5} (or $V^{-5/3}$), as predicted by crystal-field theory and experimentally observed in several transition metal halides with the perovskite structure (refs. 1-3). Such differences have also been observed in low-dimensional systems (ref. 3) where the variation of $V^{1/3}$ and R is different. An exponent close to 5 is found for the variation of Δ with R_{Co-Cl} instead of V, although reliable data of R_{Co-Cl} from XRD were possible in the 0-14 GPa range.

1. Rodríguez F. & Moreno M., *J. Phys. Chem.* **84**, 692 (1986).
2. Hernández, D., Rodríguez, F., Moreno, M., & Güdel, H. U. *Physica B* **265,** 186 (1999).
3. Nataf, L. *et al.*, *Phys. Rev. B* **89**, 115120 (2014).

$CoCl_2$ (S.G. $R\bar{3}m1$)	a (Å)	c (Å)	z_{Cl}	V_{eq}(Å^3)
US+PW	3.551	20.773	0.270	76.412
US+PW+VDW	3.512	17.445	0.257	63.088
Exptl.	3.54	17.43	0.25	63.197

Pressure range	Murnaghan's Equation of State: B_0 (GPa)	B'	V_0 (Å^3/Co)
$P < 14$ GPa	29.6 ± 1.8	5.5 ± 0.7	31.34 ± 0.15
$P > 14$ GPa	140 ± 30	1 ± 1	27.3 ± 0.5

Absorption band assignment (O_h)	Peak position at ambient conditions in $CoCl_2$: Observed (eV)	Calculated (eV)	Observed (cm^{-1})	Calculated (cm^{-1})
$^4T_1{}^a$ (F)→ $^4T_2$ (F)	0.79	0.77	6400	6220
→ $^4A_2$ (F)	1.67	1.66	13440	13390
→ $^2T_2$ (G)	-	1.90	-	15300
→ $^2T_1$ (G)	-	1.93	-	15600
→ $^4T_1{}^b$ (P)	2.10	2.12	16930	17070
→ $^2T_1$ (H)	2.22	2.21	17920	17820
→ $^2A_1$ (G)	2.35	2.36	18960	19000

Crystal-field parameters at ambient conditions

B = 0.097 eV (785 cm^{-1}) C = 0.399 eV (3220 cm^{-1}) C/B = 4.1

Δ = 0.873 eV (7040 cm^{-1})

σ = 0.014 eV (120 cm^{-1})

Pressure dependence linear fits	$E(P) = E_0 + \left[\frac{\partial E}{\partial P}\right] P$ (eV, GPa)	(cm^{-1}, GPa)
$^4T_1{}^a$ (F)→ $^4T_2$ (F)	$0.82 + 0.00115\ P$	$6630 + 92.6\ P$
→ $^4A_2$ (F)	$1.67 + 0.0026\ P$	$13440 + 209\ P$
→ $^2T_1$ (G)	$1.965 - 0.0055\ P$	$15850 - 44.3\ P$
→ $^2T_2$ (G)	-	
→ $^4T_1{}^b$ (P)	$2.10 + 0.0068\ P$	$16930 + 55\ P$

Pressure dependence of crystal-field parameters		
B	$0.097 - 0.0003\ P$	$785 - 2.4\ P$
Δ	$0.873 + 0.013\ P$	$7040 + 105\ P$
Δ/B	$9.0 + 0.17\ P$	
SCO (HS↔LS)	$E[^4T_2$ (F)$] = E[^2T_1$ (G)$]$ →	P_{SCO} = 70(5) GPa

Table S1. Experimental (room temperature) and calculated (0 K) $CoCl_2$ lattice parameters and Wyckoff positions for $CdCl_2$-type (space group, $R\bar{3}m1$) at ambient pressure and Murnaghan's Equation-of-State parameters. Co^{2+} crystal-field energies at ambient conditions, and corresponding pressure shift rate are given with same colors of manuscripts Figs. 2 and 3, and Supplementary Fig. S4. Fitting parameters Δ and B and corresponding pressure rates, as well as standard deviations are included.

FIRST-PRINCIPLES THEORETICAL CALCULATIONS

VAN DER WAALS (vdW) CALCULATIONS

$CoCl_2$ ($R\bar{3}m1$)		*a* (Å)	*c* (Å)	*c/a*	z_{cl}	V_{eq} (Å^3)
EXP		3.54	17.43	4.924	0.25	63.197
PBE		3.59	18.41	5.133	0.256	68.340
PBEsol		3.50	17.22	4.926	0.253	60.772
vdW	D3	3.48	16.00	4.772	0.252	57.988
	TS	3.58	17.46	4.878	0.253	64.575
	MBD	3.54	17.18	4.850	0.253	62.187

Table S2. The results for the full lattice optimization for $CoCl_2$ in $R\bar{3}m1$ phase. Compared to experimental values (EXP), different vdW implementations of D3[1], TS[2], and MBD[3] generates small underestimation of *c/a* ratio by 3.1%, 0.9%, and 1.5% while PBEsol functional gives even better agreement (less than 0.06 % overestimation). For the vertical position of Cl atom (z_{cl}), PBEsol functional predicts similar performance with vdW corrected methods. For the equilibrium volume (V_{eq}), PBEsol functional slightly underestimates (-3.8%) compared to TS-vdW (+2.2%) and MBD-vdW (-1.6%) but performs far better than D3-vdW (+8.2%). Note that simple PBE fails in both *c/a* and V_{eq} predictions.

DFT+U SIMULATIONS: SPIN CROSSOVER AND ELECTRON DENSITY PLOTS IN $CoCl_2$

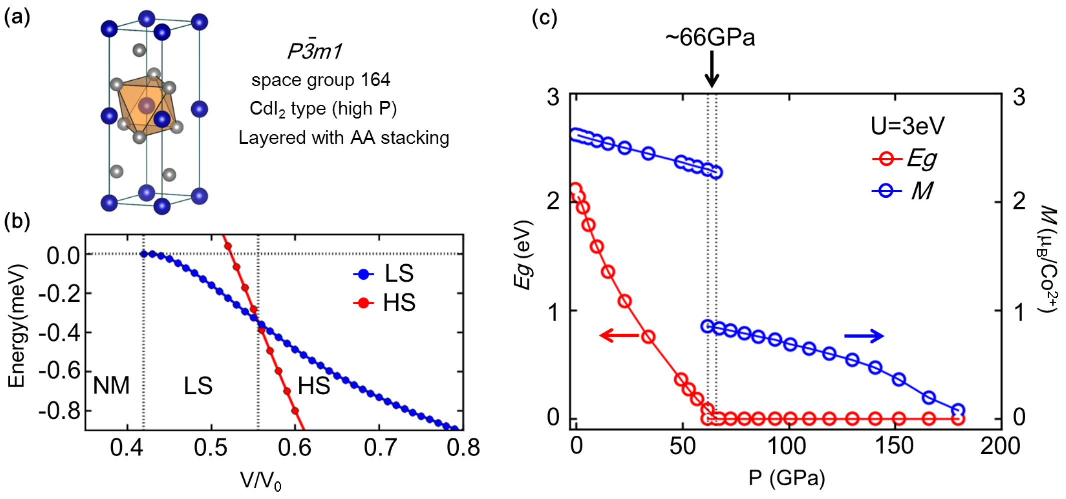

Figure S6. DFT+U calculations performed on $CoCl_2$ (space group $P\bar{3}m1$ (a)). Total energy calculations as a function of volume were performed using a Coulomb correlation energy, $U = 3$ eV. (b) for LS and HS magnetic configuration. The SCO transition is found at $V/V_0 = \sim 0.58$, corresponding to a pressure of $P = \sim 66$ GPa. The variation of the energy gap, E_g, and the magnetic moment of Co^{2+}, M, (c), derived from the electron dispersion curves and electron density (Fig. S8), respectively indicates that the SCO from HS (S = 3/2) to LS (S=1/2) takes place about 66 GPa. The energy gap closure takes place about the same pressure. It must be noted that the energy gap values derived from DFT+U are underestimated with respect to the experimental ones obtained by optical spectroscopy by about 50% (see Figs. S4 and S5), hence the slight pressure underestimation for $E_g = 0$: calculated and experimental metallization pressure is $P_{metal} = 66$ and 80 GPa, respectively.

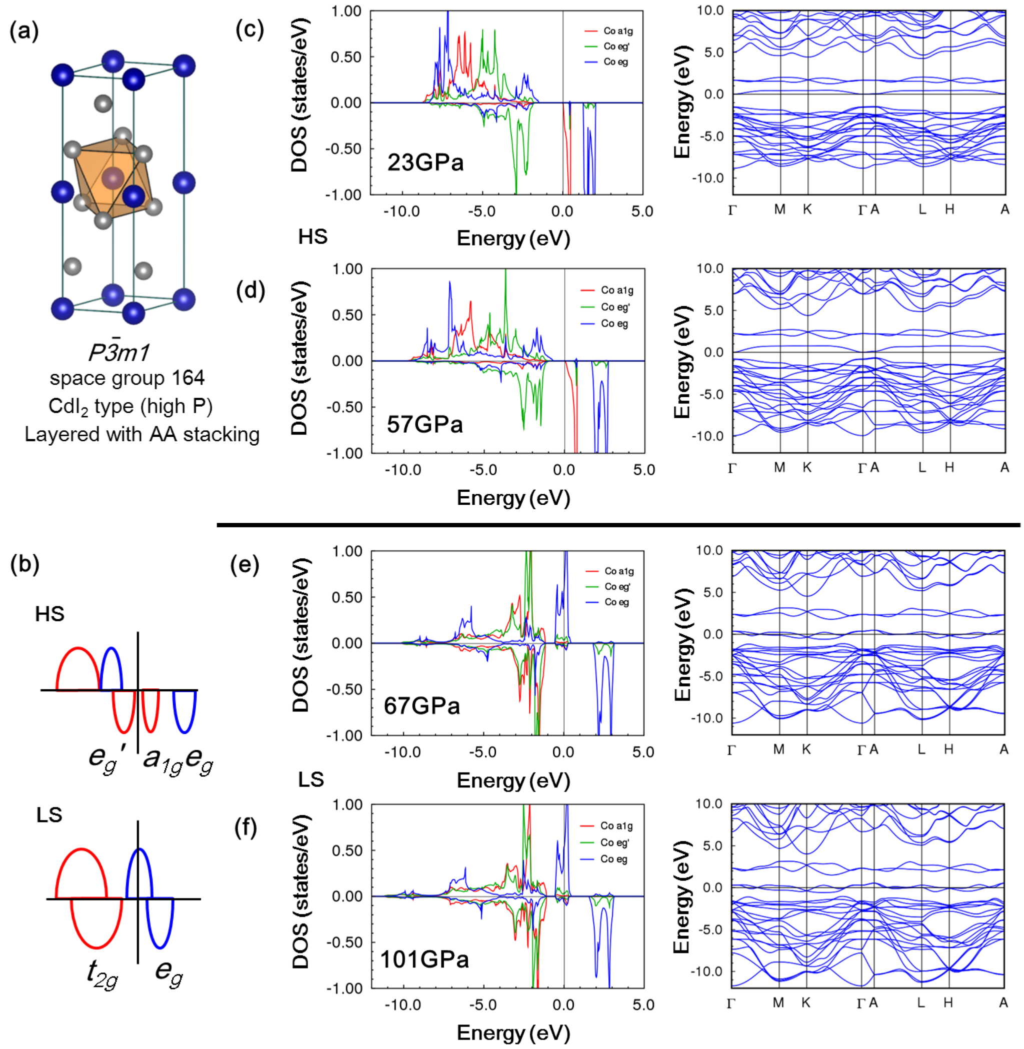

Figure S7. DFT+U (U = 3 eV) calculations performed on $CoCl_2$ (space group $P\bar{3}m1$(a)). Calculated electron density of states (DOS) projected on the Co^{2+} *3d* orbitals, and electron band dispersion curves ($E(\boldsymbol{k})$) at P = 23, 57, 67 and 101 GPa (c-f), illustrating the SCO transition at 67 GPa (right side panel). A schematic Co^{2+} *3d* bands associated with $e_{g'}$, a_{1g} and e_g orbitals (D_{3d} local symmetry); t_{2g} ($e_{g'}$, a_{1g}) + e_g in O_h, illustrates the HS and LS states. (b) Note their relative energy with respect to the Fermi level (horizontal line).

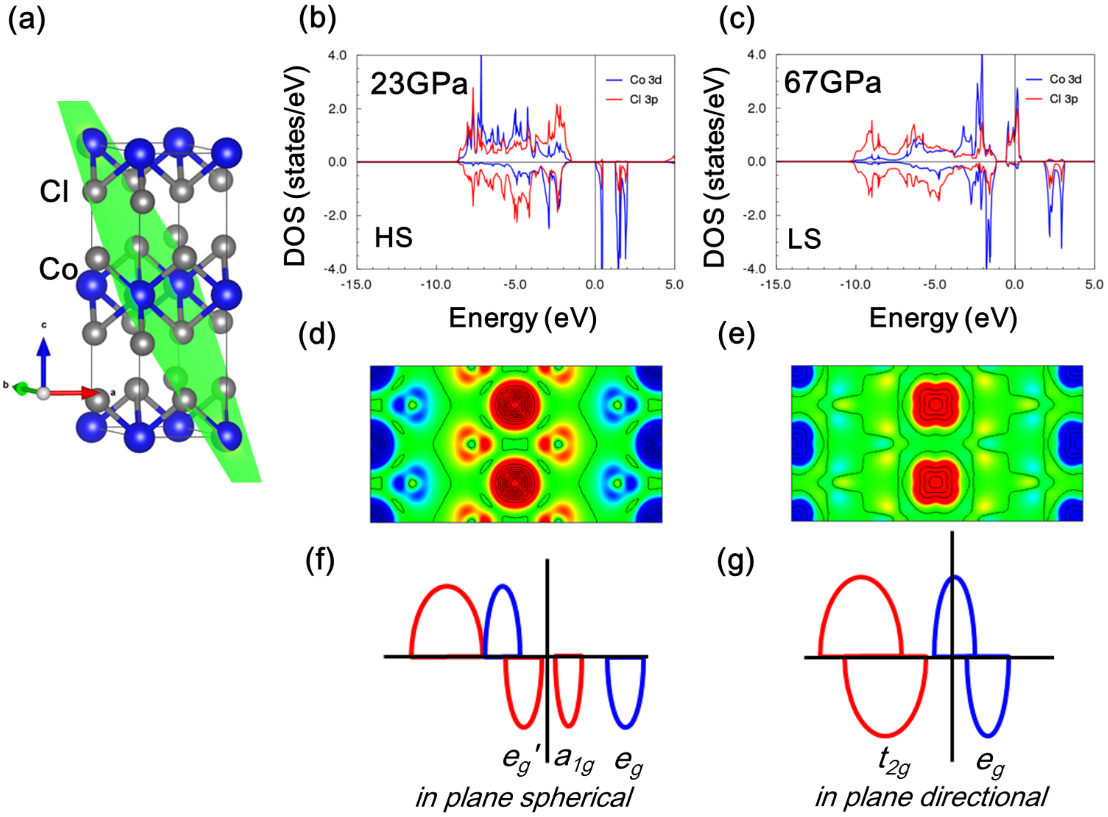

Figure S8. DFT+U (U = 3 eV) calculations performed on $CoCl_2$ (space group $P\bar{3}m1$(a)). Calculated electron DOS projected on the Co^{2+} *3d* orbitals in HS (23 GPa) and LS (67 GPa) (b,c), and corresponding spin density around the Fermi level (d,e). A schematic Co^{2+} *3d* bands associated with t_{2g} ($e_{g'}$, a_{1g}) + e_g in blue and red colors, respectively, illustrate the HS and LS states(f,g) . Note their relative energy with respect to the Fermi level (horizontal line). It must be noted that spin density in LS is significantly localized around Co^{2+} whereas it is widely delocalized around Cl- in spite the electronic Fermi levels are made of approximately same contributions from Cl^--*p* and Co^{2+}-*d* orbitals.

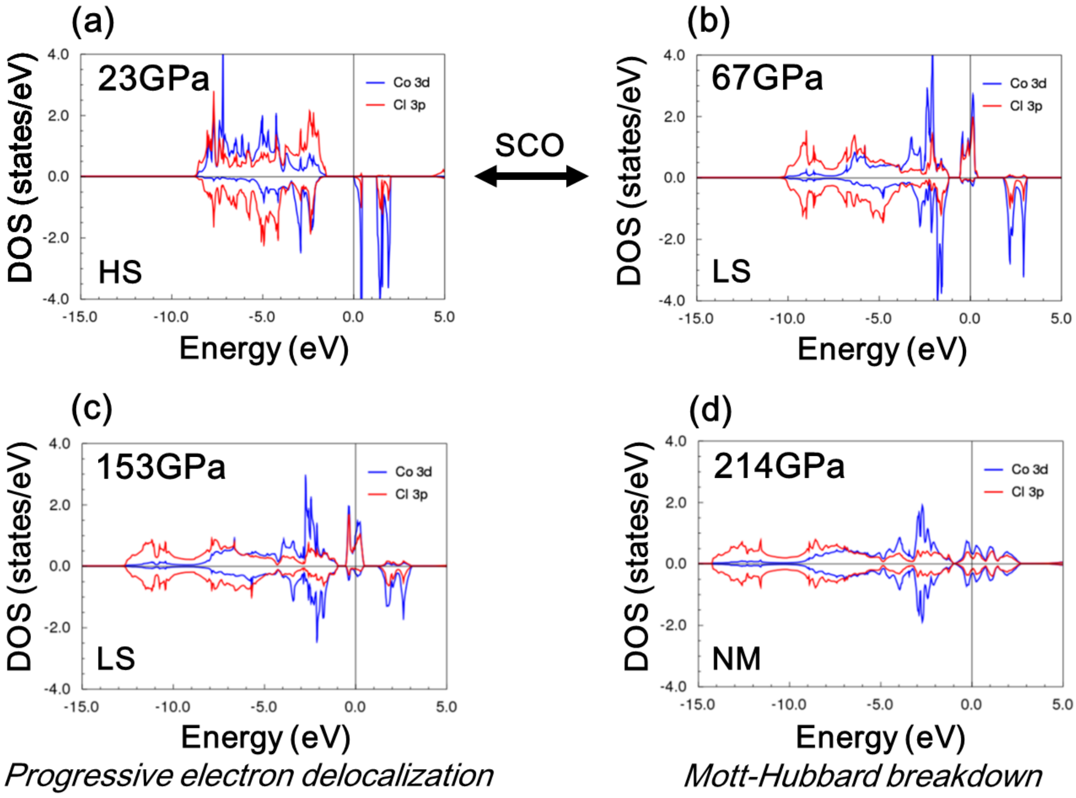

Figure S9. DFT+U (U = 3 eV) calculations performed on $CoCl_2$ (space group $P\bar{3}m1$). Calculated electron DOS projected on the Co^{2+} *3d* orbitals, and electron density around the Fermi level at different pressures: 23 GPa (HS)(a), 67 GPa (LS)(b), 101 GPa (LS: progressive *e* delocalization) (c), and 214 GPa (NM: Mott-Hubbard breakdown) (d). It must be noted that electron delocalization in LS increases with pressure, and above the gap closure ($E_g = 0$ for $P >$ 80 GPa), the electron density is still localized around Co^{2+} whereas it is almost delocalized around Cl^- in spite the electronic Fermi levels are made of approximately same contributions from Cl^--*p* and Co^{2+}-*d* orbitals. At 214 GPa, the electron density is fully delocalized. Note that above 150 GPa, calculated pressures can be overestimated since we keep the same value U = 3 eV along with this series. We expect the delocalization will decrease the effective Coulomb interactions, and decrease proper U parameter value.

Raman spectroscopy

(a)

(b)

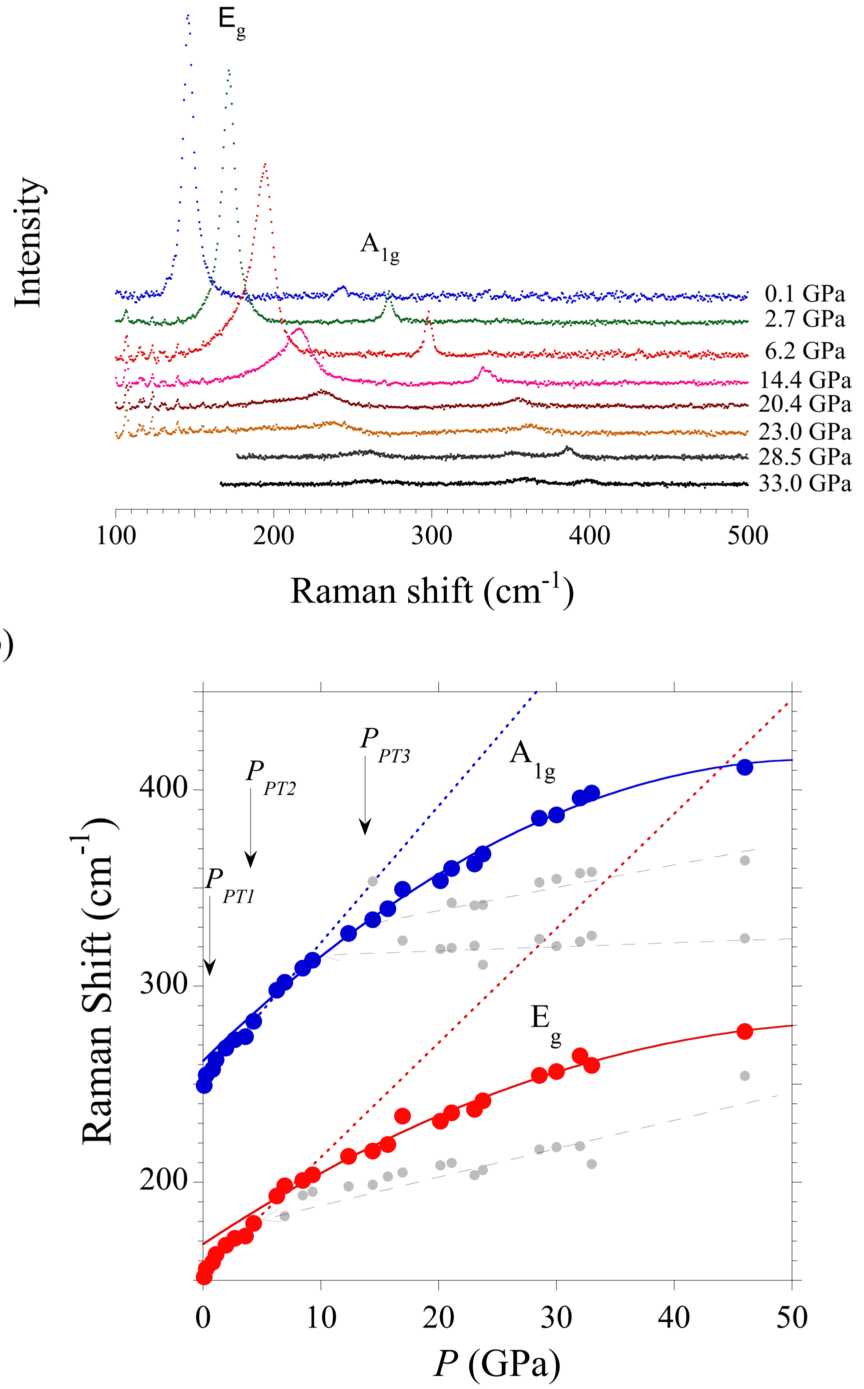

Figure S10 (a) Variation of the Raman spectrum of $CoCl_2$ with pressure showing the two Raman active A_{1g} (out-of-layer stretching) and E_g (in-layer shear) modes in the trigonal $R\bar{3}m1$ ambient pressure phase. The spectra were obtained from a single-crystal plate (90×100×35 μm^3) oriented perpendicular to *c*. The sample was excited with the 647 nm line of a Kr^+-Ar^+ laser incident along *c*. (b) Variation of the corresponding phonon frequencies with pressure. Broken lines correspond to the linear variation of $\nu(P)$ below P_{T2}, whereas above $\nu(P)$ exhibits a quadratic behaviour with pressure. Fitting parameters are collected in Table S3.

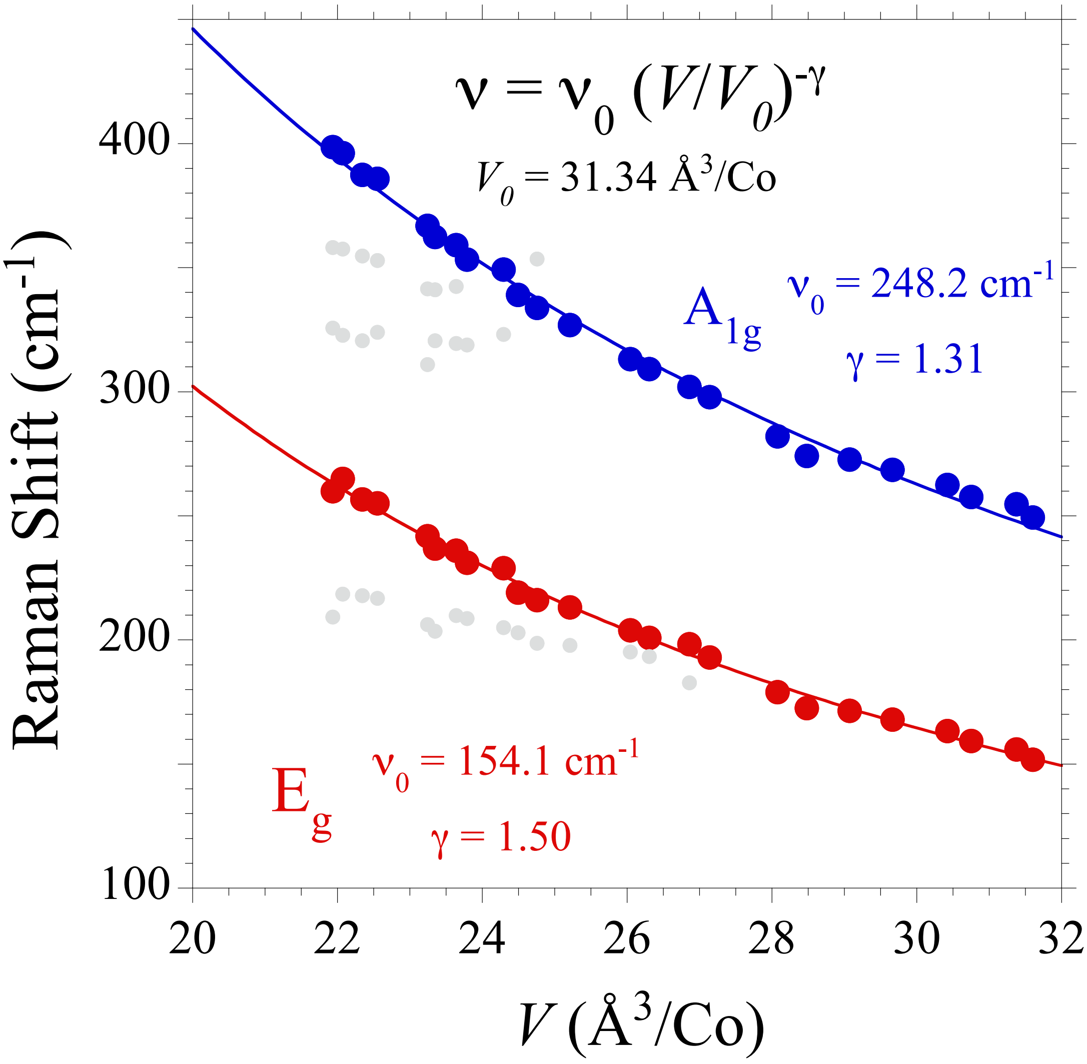

Figure S11 Variation of the Raman vibrational frequencies with the crystal volume, $\nu(V)$, in $CoCl_2$. The volume is referred to one Co atom. $\nu(V)$ was derived from $\nu(P)$ through the $CoCl_2$ Equation of State given in Figure S3. Lines correspond to the least-square fitting of experimental points to the equation: $\nu(V) = \nu_0(V/V_0)^{-\gamma}$, γ being the Grüneisen parameter of each vibrational mode; ν_0 and V_0 are the mode frequency and the volume per Co at ambient pressure, respectively.

Pressure range	Mode symmetry (Ref. 38, main text)	ν_0 (cm^{-1})	$\partial\nu/\partial P$ (cm^{-1}/GPa)	$\gamma^{(*)}$
$P < 4$ GPa	A_{1g} (out-of-plane)	252.3	7.0	0.8
	E_g (in-plane)	154.4	5.8	1.1
$P > 4$ GPa	A_{1g} (out-of-plane)	$262 + 6P - 0.06P^2$		
	E_g (in-plane)	$168.5 + 4P - 0.035P^2$		
$\nu(V)$	$\frac{\nu}{\nu_0} = \left(\frac{V}{V_0}\right)^{-\gamma}$	ν_0 (cm^{-1})	V_0 (Å^3)	γ
0 – 50 GPa	A_{1g} (out-of-plane)	248.2	31.34	1.31
	E_g (in-plane)	154.1	31.34	1.50

(*) $\gamma = (B/\nu_0)(\partial\nu/\partial P)$ $B_0 = 29.6$ GPa

Table S3 Raman active mode frequencies and corresponding pressure derivatives in $CoCl_2$. Mode assignment is made on the basis of the ambient conditions structure (Space Group $R\bar{3}m1$) following Ref. 38, main text. The Grüneisen parameters have been obtained with a bulk modulus B_0 = 29.6 GPa from the linear dependence of $\nu(P)$ in the low-pressure range (P < 4 GPa). The Grüneisen parameters for the whole pressure range (0-50 GPa) has been obtained directly by transforming $\nu(P)$ to $\nu(V)$ through the $CoCl_2$ Equation of State (Figure S3).

Electrical resistance under pressure

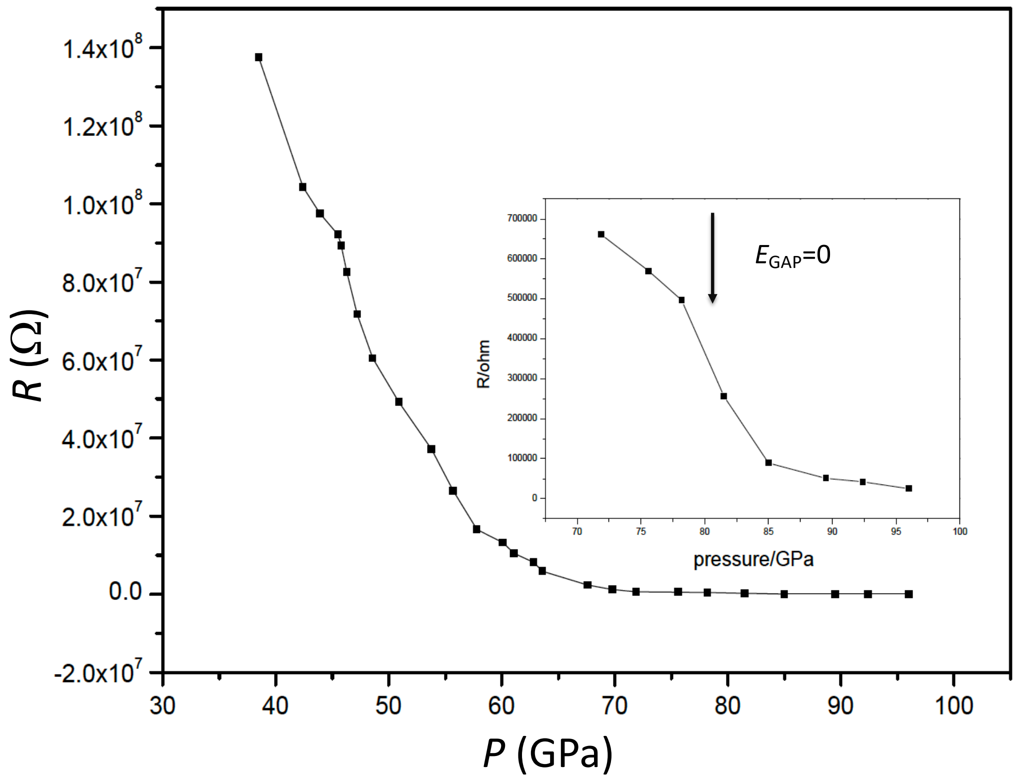

Figure S12 Electrical resistance of $CoCl_2$ in the 38-91 GPa range. *R*(*P*) decreases following an approximate exponential behaviour with pressure –semiconducting with pressure-induced E_{GAP} decrease– up to 80 GPa (metallization pressure). The inset shows a magnification of *R*(*P*) in the high-pressure range to illustrate its behaviour around the metallization pressure (E_{GAP} = 0) determined by optical spectroscopy.
